# Net energy and its establishment of prediction equations for wheat bran in growing pigs

**DOI:** 10.5713/ab.22.0001

**Published:** 2022-06-24

**Authors:** Zhiqian Lyu, Yifan Chen, Fenglai Wang, Ling Liu, Shuai Zhang, Changhua Lai

**Affiliations:** 1 State Key Laboratory of Animal Nutrition, College of Animal Science and Technology, China Agricultural University, Beijing 100193, China; 2 Guangdong HAID Group Co., Ltd., Guangzhou 511446, China; 3 College of Animal Science and Technology, Hebei Agricultural University, Baoding 071000, China

**Keywords:** Growing Pig, Heat Production, Net Energy, Prediction Equation, Wheat Bran

## Abstract

**Objective:**

The objective of this experiment was to determine the net energy (NE) value of 6 wheat bran and 1 wheat shorts by indirect calorimetry and establish the NE prediction equations of wheat bran fed to growing barrows.

**Methods:**

Forty-eight growing barrows (28.5±2.4 kg body weight) were allotted in a completely randomized design to 8 dietary treatments that included a corn-soybean meal basal diet, 6 wheat bran diets and 1 wheat shorts diet. The inclusion level of wheat bran or wheat shorts in diets is 30%.

**Results:**

The addition of wheat bran reduced the apparent total tract digestibility (ATTD) of nutrients (p<0.05). The ATTD of gross energy, crude protein (CP) and dry matter (DM) in the wheat shorts were greater than that in the wheat bran. Addition of wheat bran or wheat shorts had no effect on total heat production and fasting heat production. The NE of wheat bran was negatively correlated with neutral detergent fiber (r = −0.84; p<0.05) and acid detergent fiber (r = −0.83; p<0.05), while it was positively correlated with CP (r = 0.92; p<0.01). The NE values of wheat bran ranged from 6.79 to 8.15 MJ/kg DM, and the NE value of wheat shorts was 12.47 MJ/kg DM. The ratio of NE to metabolizable energy for wheat bran fed to growing pigs was from 66.0% to 71.7%, whereas the value for wheat shorts was 83.7%.

**Conclusion:**

The NE values of wheat bran ranged from 6.79 to 8.15 MJ/kg DM, and the NE value of wheat shorts was 12.47 MJ/kg DM. The NE value of wheat bran can be well predicted based on energy content and proximate analysis.

## INTRODUCTION

Recently, the control of pig feed costs is facing unprecedented pressure due to the price of feed ingredients continuing to rise. Cereal by-products have been proven to replace part of corn and soybean meal, thereby reducing feed costs without affecting animal growth performance [1,2]. Wheat bran and wheat shorts are by-products of the wheat milling industry produced when wheat is processed into flour for human consumption and are commonly used as feed ingredients in swine diets [3]. However, differences in variety of wheat and processing methods lead to large variations in chemical composition of wheat bran. The key to improve the efficiency for wheat bran is to accurately evaluate nutritional value of wheat bran samples. The digestible energy (DE) prediction equations for wheat bran has been established by Zhang [4], but DE system does not meet the requirements for precision feeding of pigs. The main difference between the net energy (NE) system and the DE and metabolizable energy (ME) systems is that the NE system considered the amount of heat increment during digestion and subsequent deposition of different nutrients [5,6]. Protein and dietary fiber increase heat increment while fat and starch can reduce heat increment, which means that the NE value is reduced when protein and fibre contents are higher while it is increased when more energy is provided by fat or starch. Accordingly, relative to the DE or ME system, the NE system provides a more accurate estimate of the dietary energy available to animal [5,6]. Therefore, it is necessary to determine energy values of high-fiber ingredients adopting the NE system. However, there is limited data on NE values of wheat bran or wheat shorts.

The method that uses chemical composition to predict the energy values of ingredients has been proven to efficiently evaluate available energy content of feed ingredients [7,8]. Especially for high-fiber ingredients, the large variation of its chemical composition leads to big differences in available energy values between different samples. However, to our knowledge, there is no equation for predicting the NE value of wheat bran in pigs. Therefore, the objectives of this experiment were to determine the NE value of 6 wheat bran and 1 wheat shorts by indirect calorimetry and to establish the NE prediction equations of wheat bran fed to growing barrows.

## MATERIALS AND METHODS

The experimental protocol used in this study was approved by the Institutional Animal Protection and Use Committee (IACUC) of China Agricultural University (No: AW91111202-1-1). The trial was conducted at the Feng Ning Swine Research Unit of China Agricultural University (Chengde, Hebei, China).

### Animals, diets and experimental design

Forty-eight growing barrows (Duroc×Large White×Landrace, initial body weight = 28.5±2.4 kg) were randomly allotted to 1 of 8 dietary treatments with 6 pigs per diet. Six wheat bran and 1 wheat shorts were collected to be used in the diets and the chemical compositions were shown in Table 1. Diets included a corn-soybean meal basal diet, 6 wheat bran-based diets and one wheat shorts-based diet (Table 2). Wheat bran or wheat shorts replaced 30.82% of the energy supplied by corn, soybean meal and amino acids in diets. Wheat bran samples 1, 2, and 3 were collected from Shandong province and other samples were collected from Henan province.

The trial was conducted for 8 periods because 6 open-circuit respiration chambers were available to pigs. Each experiment period lasted for 20 days including a 14-d diet adaptation period and a 6-d heat production (HP) measurement period. From d 0 to 14, pigs were fed at metabolism crates (1.4×0.7 ×0.6 m) and these metabolism crates were located in an environmentally controlled room in which the temperature was kept at 22°C±2°C. On the morning of the 14th day, pigs were transferred to the chambers for measuring the concentrations of O_2_, CO_2_ and CH_4_. On d 19, pigs were fasted. Meanwhile, the HP from 2230 h (d 19) to 0630 h (d 20) was considered as fasting heat production (FHP). To base FHP using the same time span as used for total HP, the 8-h HP was then extrapolated to a 24-h period. Pigs were fed equal sized meals twice daily at 0830 h and 1530 h and had free access to water via a low-pressure nipple drinker throughout the trial. The temperature in chambers was maintained at 22°C during the fed state and 24°C during the fasted state. The relative humidity was controlled at 70%±5%. Detailed description about the open-circuit respiration chambers were reported by Lyu et al [9].

### Sample collection

During collection period (d 15 to 19), feed refusals and spillage were collected once daily, subsequently dried and weighed. Feces were collected totally for each pig once daily at 0830 h from specialized feces trough and were immediately stored in plastic bags at −20°C.

Urine was removed at 0830 h for each pig from plastic buckets containing 50 mL of 6 *N* HCl. Subsequently, the collected urine was filtered through cotton gauze and 10% of the daily urine excretion was stored at −20°C. At the end of collection period, urine samples were thawed, and thoroughly mixed, and two sub-samples of 50 mL were saved for analysis. Urine was collected separately during fasting period for the calculation of FHP. At the end of the experiment, fecal samples were thawed, mixed, and weighed. The sub-samples were oven-dried for 72 h at 65°C. The feed and fecal samples were ground through a 1-mm screen prior to chemical analysis.

### Chemical analyses

Ingredients, diets and feces were analyzed for dry matter (DM; method 934.01 [10]), ether extract (EE; Thiex et al [11]), ash (method 942.05 [10]). Nitrogen in samples of all diets, urine, and feces were analyzed (method 984.13 [10]) using an apparatus (Foss Kjeltec 2100; Foss Kemao Inc., Beijing, China). The crude protein (CP) content was calculated as nitrogen×6.25. The gross energy (GE) in the ingredients, diets, feces, and urine samples was determined using an isoperibol calorimeter (Parr 6300 Calorimeter, Moline, IL, USA). The neutral detergent fiber (NDF) and acid detergent fiber (ADF) contents in ingredients, diets, and feces samples were determined using a fiber analyzer (model A220 fiber analyzer; Ankom Technology, Macedon, NY, USA) following a modification of the procedure by Van Soest et al [12]. Total dietary fiber (TDF) and insoluble dietary fiber (IDF) in ingredients were analyzed by using a combination of enzymatic and gravimetric procedures [13]. The concentration of soluble dietary fiber (SDF) in the ingredients was calculated as the difference between TDF and IDF.

### Calculations

Energy lost as methane was calculated using the 39.5 kJ/L conversion factor [14]. The ME content in diets was calculated by subtracting urine energy and methane energy from DE. The apparent total tract digestibility (ATTD) of GE and nutrients were calculated according to Adeola et al [15] using the following equation:


$$\text{ATTD}=[({\text{F}}_{\text{i}}-{\text{F}}_{\text{f}})/{\text{F}}_{\text{i}}]\times 100\%$$

Where F_i_ is the total intake of energy (kJ) or nutrients (g) corresponding to each collection period, and F_f_ is the total fecal output of energy (kJ) or nutrients (g) corresponding to each collection period.

Total HP or FHP of pigs was calculated for each day by gas exchange volumes and urinary N loss according to Brouwer et al [14] using the following equation:


$$\text{HP }(\text{kJ})=16.18\times {\text{O}}_{2}\;(\text{L})+5.02\times {\text{CO}}_{2}\;(\text{L})-2.17\times {\text{CH}}_{4}\;(\text{L})-5.99\times \text{urinary N }(\text{g})$$

The respiratory quotient was calculated as the ratio between CO_2_ production and O_2_ consumption. Retained energy (RE) in pigs fed different diets was calculated according to the following equation [16]:


RE (kJ/kg DM)=(ME intake (kJ/d)-HP (kJ/d))/DM intake (kg/d)

Retained energy as protein (RE_P_) was calculated as N retention (g)×6.25×23.86 (kJ/g). Retained energy as lipid was calculated as the difference between RE and RE_P_.

Net energy content in diets was calculated according to Noblet et al [16] using the following equation:


NE (kJ/kg DM)=(RE (kJ/d)+FHP (kJ/d))/DM intake (kg/d)

Firstly, the DE, ME, and NE of the corn and soybean meal mixture were calculated by DE, ME, and NE of the basal diet dividing 97.0% (the addition ratio of corn, soybean meal, and AA in the basal diet). The average GE, DE, ME, and NE values of the corn and soybean meal and AA mixture obtained for the basal diet was assumed to be same as in the other experimental diets. Secondly, the calculated GE, DE, ME, and NE values (GEc, DEc, MEc, and NEc) of wheat bran were calculated using the difference method [15]. During this calculation, the mean GE, DE, ME, or NE value in the basal diet (MJ/kg of DM) is used to calculate GEc, DEc, MEc, and NEc of wheat bran. The DE/GE, ME/DE, and NE/ME ratios for test ingredients could then be calculated and used to estimate the final DE, ME, and NE values from these calculated GE, DE, ME, and NE values and used to estimate the final DE, ME, and NE values as the product of measured GE and DE/GE for DE, measured GE and DE/GE and ME/DE for ME and measured GE and DE/GE, ME/DE, and NE/ME for NE. Lastly, the DEc/GEc, MEc/Dec, and NEc/MEc ratios could be calculated for each wheat bran sample. The values of GEm were measured ones in the laboratory using Isoperibol Calorimeter. Then the final DE, ME, and NE values for the target wheat bran sources can be estimated as: DE = GEm×DEc/GEc; ME = DE×MEc/DEc; NE = ME×NEc/MEc. Because there is only one estimated energy value for each wheat bran source, the statistical analysis cannot be conducted among energy values of wheat bran sources [17].

### Statistical analyses

All data for the experiment were analyzed by general linear model using the MIXED procedure of SAS (SAS Institute Inc., Carry, NC, USA). The model included the dietary treatments as main effects and period and chamber as random effects. Mean values for these data were separated by the LSMEANS statement with Tukey’s adjustment. To eliminate the effect of ME intake, the HP data were adjusted for each collection period by covariance analysis for ME intake of 2,254 kJ/kg BW^0.60^/d (mean value for the experiment; [17]). Tukey’s multiple range test was used to test the significance of differences among main effects, and differences were considered significant if p<0.05. PROC CORR in SAS was run to obtain the relationship between energy content and chemical composition using 6 wheat bran samples. Prediction equations for NE in the 6 wheat bran samples were developed using PROC REG of SAS. First, a simple regression on each of the considered explanatory variables with the dependent variable was performed, and then the regression equation corresponding to the explanatory variable that contributes the most to the dependent variable was used as the basis, and the remaining explanatory variables were gradually introduced into this procedure. After a stepwise regression, the explanatory variables that remained in the model were both significant and did not have severe multicollinearity. The R^2^, root mean square error, and Akaike information criterion and Bayesian information criterion were used as the selection criteria for the best fit equations. Equations with the greatest R^2^ and the least root mean square error were proposed to be the best fit.

## RESULTS

### Chemical composition of ingredients

The analyzed chemical compositions (DM basis) of ingredients are shown in Table 1. The coefficient of variation (CV) for EE, starch, NDF, ADF, IDF, SDF, and TDF was more than 10%. The concentration of CP, TDF and starch averaged 18.2% (from 17.1% to 19.8%), 44.0% (from 35.8% to 50.5%) and 17.2% (from 12.8% to 20.8%), respectively. The starch content of wheat shorts was 31.9%, which was 84.7% greater than mean value in wheat bran. The TDF content in wheat shorts was 21.5%, which was about half of the TDF content in wheat bran. The SDF content was greater in wheat shorts, while wheat bran contained more IDF content.

### Nutrients digestibility and nitrogen balance for diets

As shown in Table 3, there were no significant differences for the ATTD of nutrients among the six wheat bran diets. The ATTD of DM and OM in wheat shorts diet was greater (p<0.05) than that in wheat bran diets 1, 3, and 5, but there were no differences for the ATTD of GE, CP, NDF, and ADF between wheat bran diets and wheat shorts diet. The ATTD of DM, GE, and OM in basal diet was greater (p<0.05) when compared with wheat bran diets 1 to 5. The addition of wheat shorts did not impact the ATTD of nutrients when compared with the basal diet. The nitrogen retention value averaged 26.8 g/d and was not affected by supplementation of wheat bran or wheat shorts.

### Energy balance and energy value for experimental diets

No differences were observed for HP and energy retention values among dietary treatments (Table 4). The ratio of NE to ME for wheat bran diets ranged from 77.5% to 82.5% with an average of 80.1%, and the value for wheat shorts diet was 83.3%. There were no differences for the DE, ME, and NE contents among the six wheat bran diets. The NE value in wheat shorts diet was greater (p<0.01) than that in wheat bran diets 1, 4, and 5. The NE value in basal diet was greater (p<0.05) than that in wheat bran diet. There were no differences for the NE value between basal diet and wheat shorts diet.

### Nutrient digestibility of nutrients and energy contents for ingredients

The ATTD of nutrients and DE, ME, and NE contents of test ingredients are presented in Table 5. The GE and CP digestibility in pigs fed wheat bran diets ranged from 57.1% to 70.8% and 52.0% to 73.2%, respectively. In contrast, the GE and CP digestibility in wheat shorts were greater than the mean value in wheat bran (83.1% vs 62.0% and 76.2% vs 64.9%, respectively). The NE to ME ratio in wheat shorts (average 83.7%) was greater than that in wheat bran (66.0% to 71.7%). The NE content in wheat bran ranged from 6.79 to 8.15 MJ/kg DM, and the value in wheat shorts was 12.47 MJ/kg DM. The mean NE content of wheat bran determined using the indirect calorimetry method were close to the predicted values (relative error ranged from −7.4% to 3.5%), whereas the NE value of wheat shorts measured using the IC method was greater than that predicted by 19.3%.

### Correlation analysis and net energy prediction equations for wheat bran samples

Results for correlation analysis between chemical composition and DE, ME, and NE of the 6 wheat bran samples tested are shown in Table 6. The NDF content was negatively correlated with CP (r = −0.83; p<0.05) and starch content (r = −0.94; p<0.01). The TDF content was negatively correlated with starch content (r = −0.90; p<0.01) and positively correlated with NDF (r = 0.95; p<0.01), ADF (r = 0.93; p<0.01), and IDF (r = 0.98; p<0.01) content. The NE value was positively correlated with CP (r = 0.92; p<0.01), DE (r = 0.93; p<0.01), and ME content (r = 0.92; p<0.01) and negatively correlated with NDF content (r = −0.84; p<0.05). The NE to ME ratio was negatively correlated with ADF (r = −0.81; p< 0.05), TDF (r = −0.79; p<0.05), and ash content (r = −0.78; p<0.05) and positively correlated with starch (r = 0.83; p<0.05).

The NE regression equations for wheat bran samples are presented in Table 7. The CP was the first predictor of NE content (R^2^ = 0.85; p<0.05), but the accuracy of the equations was improved if GE was included in the prediction equation (R^2^ = 0.94; p<0.01). Metabolizable energy had a high correlation with NE and, therefore, the ME content can be used as a single predictor in the NE prediction equations (R^2^ = 0.85; p<0.01). When the GE was included in the equation, the accuracy of the equation was improved (R^2^ = 0.94; p<0.05). Otherwise, the NDF (R^2^ = 0.71; p<0.05) or ADF (R^2^ = 0.68; p<0.05) can be used as a single predictor to predict the NE value of wheat bran.

## DISCUSSION

### Chemical composition of ingredients

The wheat bran samples used in this study came from two major producing regions, Shandong and Henan province in China. The origin, variety, growing environment of raw wheat and differences in processing conditions lead to variable chemical composition of wheat bran [18]. The wide variations in the chemical composition of wheat bran were consistent with results from Zhang [4] and Huang et al [19], who reported that the CV of starch, ADF, and EE in wheat bran was more than 10%. The average values of CP, NDF and ash were greater than those reported by the NRC [20] and Stein et al [21], while the starch content was less. Wheat bran is the end by-products mainly produced from wheat flour industry and made up of seed coat, pericarp, nucellar epidermis, and aleurone layer [22]. Wheat shorts includes aleurone layer, endosperm and a small amount of fine wheat bran, which is a by-product of refined wheat flour processing that removes wheat bran, germ and qualified wheat flour [23]. The wheat shorts contained more starch content and less fiber components than wheat bran samples, which was due to differences in processing techniques and human demands for wheat flour [23]. The starch and NDF contents in wheat shorts were within the range of values reported by Huang et al [24], who reported the CV values of starch and NDF content for wheat shorts were more than 10%.

### Nutrient digestibility, nitrogen balance and energy metabolism for diets

The nutrient digestibility in diets is affected by dietary characteristics especially for the fiber concentrations. The addition of fiber-rich ingredients (i.e., wheat bran) to diets decreased the ATTD of nutrients, thus affecting negatively the energy available to pigs [6].

In the current study, pigs retained an average of 26.8 g of nitrogen per day that was not affected by diets and was consistent with the results of Lyu et al [25] and Liu et al [26], who reported that the maximum amount of nitrogen deposited by growing barrows was about 23.3 to 28.7 g/d. The results for the effects of dietary fiber on HP is controversial, mainly because how dietary fiber affects activity-related HP is still inconclusive [27–29]. The HP of animals is not only related to the physiological state of the animals itself and diet structure, but also related to the experimental environment. The FHP determined by the indirect calorimetry technology is considered to be equivalent to the NE maintenance requirement of pigs, the values of FHP account for 30% to 35% of ME intake [30,31]. A recent study indicated that FHP had a weak correlation with dietary composition, but it was strongly affected by feed intake before fasting period [32]. Zhang et al [33] found that FHP increased as feeding level increased. The NE to ME ratio was affected by the dietary composition and the method for FHP measurement. Based on results from current and previous studies [16], the dietary fiber concentration in diets was negatively correlated with the NE to ME ratio while starch was positively correlated with the ratio value. In addition, The FHP value was generally greater than the value determined by regression method [33]. Accordingly, the NE to ME ratio was greater than the value reported by Noblet et al [16], who found NE to ME ratio for diets ranged from 69.0% to 77.2% when 750 kJ/kg BW^0.6^ obtained by regression method was used as FHP value. In addition, differences in experimental conditions, feeding strategies, genetics and body weight of pigs, which influence energy expenditure, growth, and body composition, may lead to variation in the ratio of NE to ME of diets [31].

### Energy utilization and energy values of ingredients

The ATTD of GE in wheat bran ranged from 57.1% to 70.8%, with an average of 62.0%, which was within the range of values (mean 61.3%; range from 48.2% to 71.3%) reported by Zhang [4]. The ATTD of CP in wheat bran is much less than the values in wheat and wheat shorts, which prevents massive use of wheat bran in piglet feed. In addition, the high concentrations and poor digestibility for fiber component in wheat bran led to restrictions on the proportion of wheat bran added to the diet. Most nutrients in wheat shorts were easier to digest by pigs than those in wheat bran, which was confirmed by Huang et al [19].

The NE to ME ratio in fiber-rich ingredients ranged from 65.3% to 83.7% with an average value of 72.9% based on published data [9,17]. In the current study, the NE to ME ratio for wheat bran is similar to results for other fiber-rich ingredients such as soybean hulls, sugar beet pulp and palm kernel expellers. The greater NE to ME ratios in the wheat shorts than in the wheat bran can be explained by the differences in efficiencies of ME utilization between nutrients with the highest values for fat (90%) and starch (82%) and the lowest (60%) for dietary fiber and CP [16]. The DE, ME, and NE values in NRC [18] were 11.6, 11.1, and 7.9 MJ/kg DM, which were within the range of values determined in the current study. The DE, ME, and NE values for wheat shorts samples were greater than values recommended by NRC [20], which was attributed to less fiber content of wheat shorts samples used in the current study. Meanwhile, it also indicted that nutritional value of different samples varies greatly and a single energy value cannot accurately estimate the energy values of wheat bran or wheat shorts samples. Huang et al [19] reported that DE and ME values in wheat bran (n = 5) ranged from 11.7 to 12.7 MJ/kg DM and from 10.4 to 11.3 MJ/kg DM, respectively, while these measured values in current study had a larger variation range. The DE and ME values for wheat shorts were within the range of values reported by Huang et al [19], who found that DE and ME in wheat shorts (n = 5) ranged from 14.2 to 16.4 MJ/kg DM and from 12.7 to 14.5 MJ/kg DM, respectively. There are limited data on NE content for wheat bran or wheat shorts. Lyu et al [25] and Liu et al [26] determined the NE content of wheat bran in growing pigs were 7.47 and 7.78 MJ/kg DM, respectively which was close to the measured mean value in the current study. The mean NE value of wheat bran determined by indirect calorimetry was close to the predicted NE values in prediction equations from Noblet et al [16]. However, a big difference was observed between the measured NE values and predicted NE value in wheat shorts. It shows that prediction equations do not necessarily apply to all ingredients [34]. In the current study, NE values of 6 wheat bran samples fed to growing pigs were determined and will provide a comprehensive reference for the reasonable use of wheat bran.

### Correlation analysis and net energy prediction equations

Previous studies reported the correlation relationship between chemical composition and DE or ME content in wheat co-products samples. The starch content was negatively correlated with NDF content in wheat co-products [19]. Meanwhile, there was a negative correlation between fiber content (NDF or ADF) and available energy values (DE or ME) in wheat co-products. Similar results were observed on other ingredients by Chen et al [35] who reported that NDF and ADF concentrations was negatively correlated with available energy values (DE or ME) in flaxseed expellers samples. In addition, the CP content was positively correlated with the energy values of wheat bran, which was consistent with results by Huang et al [19]. Similarly to our results, Shi et al [36] reported that EE and GE of corn germ meal had a weak correlation with DE or ME value. Meanwhile, in this study, EE had a weak correlation with most nutrients (CP, NDF, ADF, starch, and ash). Yang et al [22] studied the correlation relationships between nutrients for wheat bran, and found that the GE and EE were not correlated with DE or ME values. In addition, the EE concentrations of wheat bran samples do not seem to be high and variable enough to significantly influence on energy values. The results for correlation between NE to ME ratio and other nutrients (starch, ADF, TDF, and ash) show that starch contributes to the improvement of NE to ME ratio in wheat bran, while fiber and ash decrease the value. To our knowledge, there were limited data on the correlation relationship between chemical composition and NE content for wheat bran, although previous studies have reported a decreased NE concentration when high inclusion levels of wheat bran were added to diets [37].

The DE and ME prediction equations for wheat co-products in growing pigs have been established by Huang et al [17]. These equations indicted that NDF content was the first predictor for predicting DE or ME of wheat co-products. In addition, Zhang [4] established the DE prediction equations for wheat bran and found that the starch content was the first predictor for predicting DE values of wheat bran. The establishment of prediction equations for ingredients depends on variety, source, chemical characteristics, digestibility of nutrients and GE, number of samples and interactions between these factors [19,35]. In the current study, the CP content was the first predictor and NDF was the second predictor for predicting NE values of wheat bran, which indicted that the best predictor of the equation depended on the selected ingredient samples. The best fit equation to predict NE values of wheat bran was NE = −20.55+1.20×GE+0.47×ME based on statistical criterion.

## CONCLUSION

The NE to ME ratio for wheat bran fed to growing pigs ranges from 66.0% to 71.7%. The NE values of wheat bran in growing pigs range from 6.79 to 8.15 MJ/kg DM, and the NE values of wheat shorts is 12.47 MJ/kg DM. Users can choose the appropriate equation according to the measured chemical composition of wheat bran, or better, use the mean value of the predicted values.

## Figures and Tables

**Table 1:** Chemical composition of the experimental ingredients (%, DM basis)

Items^1)^	Wheat bran						Mean	CV^2)^ %	Wheat shorts
	
	1	2	3	4	5	6			7
GE (MJ/kg)	18.72	19.15	19.07	19.00	19.02	19.24	19.03	0.9	19.20
DM	87.95	87.79	86.92	87.88	87.58	88.32	87.74	0.5	89.80
CP	17.32	18.84	19.84	17.06	17.09	19.03	18.20	6.0	20.02
EE	2.13	1.97	2.87	2.70	1.82	2.15	2.27	16.8	2.67
Starch	12.81	17.04	20.81	18.71	15.92	18.15	17.24	14.3	31.85
NDF	51.01	39.63	34.23	42.39	45.44	40.06	42.13	12.1	23.25
ADF	14.47	10.44	9.01	11.28	11.95	10.74	11.32	14.8	3.73
IDF	46.88	37.86	29.73	39.64	45.88	42.20	40.37	14.2	16.93
SDF	3.56	3.79	6.01	3.56	2.17	2.45	3.59	34.5	4.57
TDF	50.45	41.65	35.75	43.21	48.05	44.65	43.96	10.7	21.49
Ash	6.66	5.78	4.82	5.66	5.62	5.24	5.63	10.0	3.20

DM, dry matter; CV, coefficient of variation; GE, gross energy; CP, crude protein; EE, ether extract; NDF, neutral detergent fiber; ADF, acid detergent fiber; IDF, insoluble dietary fiber; SDF, soluble dietary fiber; TDF, total dietary fiber.

**Table 2:** Ingredient composition and chemical composition of experiment diets

Items	Basal diet	Wheat bran diet						Wheat shorts diet
	
		1	2	3	4	5	6	7
Ingredients (%)								
Corn	71.61	49.54	49.54	49.54	49.54	49.54	49.54	49.54
Soybean meal	24.95	17.26	17.26	17.26	17.26	17.26	17.26	17.26
Wheat bran	0.00	30.00	30.00	30.00	30.00	30.00	30.00	-
Wheat shorts	-	-	-	-	-	-	-	30.00
Dicalcium phosphate	0.90	0.90	0.90	0.90	0.90	0.90	0.90	0.90
Limestone	0.90	0.90	0.90	0.90	0.90	0.90	0.90	0.90
Salt	0.35	0.35	0.35	0.35	0.35	0.35	0.35	0.35
Premix^1)^	0.50	0.50	0.50	0.50	0.50	0.50	0.50	0.50
L-Lys·HCl (78%)	0.50	0.35	0.35	0.35	0.35	0.35	0.35	0.35
DL-Methionine	0.07	0.05	0.05	0.05	0.05	0.05	0.05	0.05
L-Threonine	0.16	0.11	0.11	0.11	0.11	0.11	0.11	0.11
L-Valine	0.06	0.04	0.04	0.04	0.04	0.04	0.04	0.04
Analyzed composition (% dry matter basis)								
Gross energy (MJ/kg)	18.41	18.36	18.54	18.70	18.43	18.46	18.62	18.55
Dry matter	88.45	88.31	87.85	88.23	88.07	88.78	88.38	88.67
Crude protein	18.76	19.05	19.54	19.89	19.90	18.43	19.52	19.84
Ether extract	2.25	2.00	2.23	2.09	2.30	1.23	2.10	2.25
Neutral detergent fiber	13.43	21.83	21.59	18.83	23.05	23.91	24.26	14.56
Acid detergent fiber	4.18	6.39	6.26	5.34	6.39	6.90	6.70	3.85
Ash	4.95	6.41	5.90	5.78	6.08	5.93	5.93	5.44
Calculated composition (% as-fed basis)								
Ca	0.65	0.65	0.65	0.65	0.65	0.65	0.65	0.64
P	0.58	0.62	0.62	0.62	0.62	0.62	0.62	0.60
Lys	1.22	1.04	1.04	1.04	1.04	1.04	1.04	1.03
Met	0.33	0.31	0.31	0.31	0.31	0.31	0.31	0.30
Thr	0.78	0.72	0.72	0.72	0.72	0.72	0.72	0.70
Trp	0.18	0.19	0.19	0.19	0.19	0.19	0.19	0.18
Val	0.85	0.81	0.81	0.81	0.81	0.81	0.81	0.80

1) Premix provided the following per kilogram of complete diet: 5,512 IU vitamin A as retinyl acetate, 2,200 IU vitamin D_3_ as cholecalciferol, 30 IU vitamin E as dl-alpha-tocopheryl acetate, 2.2 mg vitamin K_3_ as menadione nicotinamide bisulfite, 27.6 μg vitamin B_12_, 4 mg riboflavin, 14 mg pantothenic acid as dl-calcium pantothenate, 30 mg niacin, 400 mg choline chloride, 0.7 mg folacin, 1.5 mg thiamin as thiamine mononitrate, 3 mg pyridoxine as pyridoxine hydrochloride, 44 μg biotin, 40 mg Mn as MnO, 75 mg Fe as FeSO_4_·H_2_O, 75 mg Zn as ZnO, 100 mg Cu as CuSO_4_·5H_2_O, 0.3 mg I as KI, and 0.3 mg Se as Na_2_SeO_3_.

**Table 3:** Effect of diet characteristics on nutrient digestibility and nitrogen balances of growing pigs fed experimental diets (n = 6)

Items	Basal diet	Wheat bran diet						Wheat shorts diet	SEM	p-value
	
		1	2	3	4	5	6	7		
Body weight (kg)	41.5	43.0	43.5	39.7	41.1	39.1	42.8	39.0	2.12	0.616
Dry matter intake (kg/d)	1.37	1.54	1.45	1.39	1.48	1.48	1.54	1.39	0.08	0.510
ATTD (%)										
Dry matter	87.82^a^	80.00^c^	80.88^bc^	78.87^c^	80.80^bc^	79.24^c^	82.40^abc^	86.63^ab^	1.37	0.003
Gross energy	88.02^a^	79.12^b^	80.00^b^	78.99^b^	79.12^b^	80.01^b^	80.11^b^	86.21^ab^	0.02	0.048
Crude protein	83.21^a^	72.80^b^	77.29^ab^	72.82^ab^	78.93^ab^	76.59^ab^	79.76^ab^	80.76^ab^	0.02	0.013
Neutral detergent fiber	62.86	54.59	57.69	49.23	58.30	57.51	63.92	63.15	0.04	0.262
Acid detergent fiber	53.87	39.14	43.16	43.22	40.08	43.09	49.50	44.67	0.04	0.156
Organic matter	89.19^a^	81.81^c^	82.80^bc^	81.11^c^	82.65^bc^	81.33^c^	84.01^abc^	88.30^ab^	0.01	0.004
UE/DE (%)	1.56	2.71	3.15	3.66	3.05	3.37	3.41	3.15	0.72	0.467
Methane energy (% of DE)^1)^	0.43	0.40	0.49	0.49	0.41	0.48	0.31	0.37	0.08	0.046
Nitrogen balance (g/d)										
Intake	41.2	47.0	44.4	44.2	46.5	43.6	47.0	44.1	2.08	0.398
Fecal output	5.4^b^	11.6^ab^	9.2^ab^	10.3^ab^	9.6^ab^	11.1^ab^	11.7^a^	8.1^ab^	1.41	0.012
Urinary output	7.7	9.5	9.1	9.1	8.7	7.6	7.9	7.5	1.19	0.831
Retention	28.2	26.0	26.2	24.9	28.2	24.9	27.4	28.4	2.45	0.618

SEM, standard error of mean; ATTD, Apparent total tract digestibility; UE, urine energy value; DE, digestible energy.

1) Methane energy (kJ) = 39.5 kJ/L×concentration of methane (L).

a–c Means with different superscript within a row are significantly different (p<0.05).

**Table 4:** Effect of diet characteristics on energy balance and energy values of growing pigs fed experimental diets (n = 6)

Items	Basal diet	Wheat bran diet						Wheat shorts diet	SEM	p-value
	
		1	2	3	4	5	6	7		
Energy balance (kJ/kg BW^0.6^/d)										
ME intake	2,311	2,228	2,133	2,138	2,240	2,320	2,306	2,355	87	0.452
THP	1,196	1,213	1,195	1,191	1,278	1,311	1,279	1,209	66	0.718
Adjusted THP^1)^	1,182	1,222	1,230	1,224	1,284	1,295	1,267	1,183	65	0.775
FHP^2)^	805	765	788	805	815	786	812	816	55	0.996
RE_P_	448	405	406	403	453	411	431	503	33	0.383
RE_L_	668	610	532	545	509	598	596	643	78	0.770
RE	1,115	1,015	938	948	962	1,009	1,027	1,147	88	0.597
Respiratory quotient										
Fed state	1.07	1.09	1.09	1.09	1.07	1.07	1.09	1.08	0.02	0.923
Fasted state	0.82	0.84	0.84	0.83	0.82	0.81	0.81	0.82	0.01	0.811
Energy utilization (%)										
ME/DE	97.8	96.8	96.5	95.8	96.5	96.2	96.2	96.8	0.03	0.400
NE/ME	83.2	80.0	80.5	82.5	79.7	77.5	80.2	83.3	0.02	0.365
Energy values, MJ/kg dry matter										
DE	16.05^a^	14.27^c^	14.69^abc^	14.59^bc^	14.55^bc^	14.74^abc^	14.81^abc^	15.82^ab^	0.32	0.010
ME	15.71^a^	13.80^b^	14.18^ab^	14.00^ab^	14.06^ab^	14.21^ab^	14.25^ab^	15.27^ab^	0.06	0.020
NE	13.08^a^	11.03^c^	11.41^bc^	11.45^bc^	11.21^c^	10.99^c^	11.43^bc^	12.74^ab^	0.21	0.003

SEM, standard error of mean; BW, body weight; ME, metabolizable energy; THP, total heat production; FHP, fasting heat production; REP, retained energy as protein; REL, retained energy as lipids; RE, retained energy; DE, digestible energy; NE, net energy.

1) Adjusted THP means the THP was adjusted for a ME intake of 2,254 kJ/kg BW^0.60^/d (mean value for the experiment) by covariance.

2) FHP was calculated using the equation for THP with gas concentrations and air flow obtained from only the last 8-h heat production measurement from d 19 to 20 (i.e., from 2200 h to 0600 h). In order to base production using the same time span as used for THP, the 8-h heat production was extrapolated to a 24-h period.

a–c Means with different superscript within a row are significantly different (p<0.05).

**Table 5:** Energy utilization and energy value of the high-fiber ingredients for growing pigs^1)^

Items	Wheat bran						Mean	Wheat shorts
	
	1	2	3	4	5	6		7
ATTD (%)								
Gross energy	57.2	62.6	70.8	57.1	60.4	63.8	62.0	83.1
CP	52.0	65.7	64.1	70.7	63.5	73.2	64.9	76.2
NDF	48.6	53.9	59.5	55.0	53.6	64.7	55.9	63.4
ADF	34.5	39.8	41.0	35.7	39.7	48.1	39.8	41.8
OM	65.1	68.4	74.6	67.9	63.6	72.3	68.7	86.3
Energy utilization (%)								
ME/DE	93.0	92.5	91.4	93.0	91.9	91.4	92.2	93.4
NE/ME	68.2	71.7	66.0	68.1	69.4	70.7	69.0	83.7
Energy values (MJ/kg DM)								
DE	10.71	11.98	13.50	10.85	11.49	12.27	11.80	15.95
ME	9.96	11.08	12.34	10.09	10.56	11.22	10.90	14.89
NE	6.79	7.94	8.15	6.87	7.33	7.93	7.50	12.47
Predicted NE_1_^1)^	6.71	7.76	8.98	7.09	7.40	7.98	7.65	10.56
Predicted NE_2_^2)^	6.61	7.58	8.63	6.95	7.17	7.69	7.44	10.35
Relative error^3)^ (%)	2.0	3.5	−7.4	−2.1	0.6	1.2	2.8	19.3

The number of observations is 6.

ATTD, apparent total tract digestibility; CP, crude protein; NDF, neutral detergent fiber; ADF, acid detergent fiber; OM, organic matter; ME, metabolizable energy; DE, digestible energy; NE, net energy; DM, dry matter.

1) Predicted NE_1_ (MJ/kg DM) = 0.700×DE (MJ/kg DM)+[(16.1×ether extract (%)+4.8×Starch (%)–9.1×CP (%)–8.7×ADF (%)]/1,000×4.184.

2) Predicted NE_2_ (MJ/kg DM) = 0.726×ME (MJ/kg DM)+[(13.3×EE (%)+3.9×Starch (%)–6.2×CP (%)–8.3×ADF (%))]/1,000×4.184.

3) Relative error = [Determined NE– (Predicted NE_1_+Predicted NE_2_)/2]/[(Predicted NE_1_+Predicted NE_2_)/2].

**Table 6:** Correlation coefficients between chemical characteristics and energy values of the experimental ingredients

Items	GE	CP	EE	Starch	NDF	ADF	IDF	SDF	TDF	Ash	DE	ME	NE	NE/ME
GE	1.00													
CP	0.60	1.00												
EE	−0.01	0.33	1.00											
Starch	0.69	0.62	0.69	1.00										
NDF	−0.76	−0.83^*^	−0.53	−0.94^**^	1.00									
ADF	−0.80	−0.76	−0.47	−0.94^**^	0.99^**^	1.00								
IDF	−0.55	−0.87^*^	−0.68	−0.87^*^	0.95^**^	0.90^*^	1.00							
SDF	−0.08	0.59	0.76	0.51	−0.57	−0.49	−0.78	1.00						
TDF	−0.55	−0.77	−0.69	−0.90^*^	0.95^**^	0.93^**^	0.98^**^	−0.78	1.00					
Ash	−0.76	−0.68	−0.48	−0.93^**^	0.91^*^	0.92^**^	0.80	−0.36	0.80	1.00				
DE	0.61	0.92^**^	0.37	0.73	−0.88^*^	−0.84^*^	−0.88^*^	0.59	−0.82^*^	−0.84^*^	1.00			
ME	0.60	0.93^**^	0.39	0.73	−0.88^*^	−0.85^*^	−0.89^*^	0.62	−0.84^*^	−0.83^*^	0.99^**^	1.00		
NE	0.79	0.92^**^	0.09	0.63	−0.84^*^	−0.83^*^	−0.78	0.36	−0.72	−0.76	0.93^**^	0.92^**^	1.00	
NE/ME	0.50	0.52	0.13	0.83^*^	−0.75	−0.81^*^	−0.73	0.08	−0.79^*^	−0.78^*^	0.78^*^	0.81^*^	0.93^**^	1.00

The number of observations is 6. The correlation coefficients are developed only using wheat bran sources.

GE, gross energy; CP, crude protein; EE, ether extract; NDF, neutral detergent fiber; ADF, acid detergent fiber; IDF, insoluble dietary fiber; SDF, soluble dietary fiber; TDF, total dietary fiber; DE, digestible energy; ME, metabolizable energy; NE, net energy.

* p<0.05,

** p<0.01.

**Table 7:** Stepwise regression equations for prediction of net energy content of wheat bran fed to growing pigs

Items	Prediction equations^1)^	R^2^	RMSE^2)^	AIC^3)^	BIC^4)^	p-value
1	NE = −21.65+1.20GE+0.35×CP	0.94	0.25	−14.96	−18.96	<0.01
2	NE = −0.80+0.46×CP	0.85	0.19	17.96	−23.96	<0.01
3	NE = 11.16−0.09×NDF	0.71	0.35	10.91	−7.05	<0.05
4	NE = 10.51−0.27×ADF	0.68	0.37	−10.38	−6.68	<0.05
5	NE = −20.55+1.20×GE+0.47×ME	0.94	0.19	−18.18	−11.86	<0.05
6	NE = 0.77+0.62×ME	0.85	0.25	15.01	−13.30	<0.01

RMSE, root mean square error; AIC, Akaike information criterion; BIC, Bayesian information criterion; NE, net energy; GE, gross energy; CP, crude protein; NDF, neutral detergent fiber; ADF, acid detergent fiber; ME, metabolizable energy.

1) The value of the energy and chemical composition in the equations as dry matter basis. These prediction equations were established using 6 sources of wheat bran.
